# Decreased metallothionein-3 expression in the human spinal cord is a common feature of amyotrophic lateral sclerosis and multiple sclerosis

**DOI:** 10.1038/s41598-025-31283-9

**Published:** 2026-03-04

**Authors:** Adam P. Gunn, James B. W. Hilton, Soumya Mukherjee, Jeffrey R. Liddell, Stephen W. Mercer, Catriona A. McLean, Colin L. Masters, Peter J. Crouch, Blaine R. Roberts

**Affiliations:** 1 The Florey Institute of Neuroscience and Mental Health, The University of Melbourne, Parkville, Melbourne, VIC 3052 Australia; 2 Department of Microbiology and Immunology, The University of Melbourne, Parkville, Melbourne, VIC 3010 Australia; 3 Department of Anatomy and Physiology, The University of Melbourne, Parkville, Melbourne, VIC 3010 Australia; 4 Centre for Muscle Research, The University of Melbourne, Parkville, Melbourne, VIC 3010 Australia; 5 Monash Micro Imaging, The Hudson Institute of Medical Research, Clayton, Melbourne, VIC 3800 Australia; 6 Department of Neurology, Washington University in St Louis, St. Louis, MO 63110 USA; 7 Department of Anatomical Pathology, Alfred Hospital, Prahran, Melbourne, VIC 3004 Australia; 8 Department of Biochemistry, Emory University, Atlanta, GA 30322 USA; 9 Department of Neurology, Emory University, Atlanta, GA 30322 USA

**Keywords:** Amyotrophic lateral sclerosis, Multiple sclerosis, Copper, Metallothioneins, Metallothionein-3 (MT3)., Amyotrophic lateral sclerosis, Multiple sclerosis

## Abstract

Amyotrophic lateral sclerosis (ALS) and multiple sclerosis (MS) are devastating adult-onset neurological diseases with distinct pathological manifestations. Although growing evidence implicates copper dysregulation in both diseases, our understanding of this connection remains incomplete. In this study, we investigated metallothionein-3 (MT3), which is a key regulator of copper metabolism in the central nervous system (CNS), and its relationship to copper in the ALS and MS spinal cord. Using combined quantitative mass spectrometry approaches and immunohistochemical observations, we found a significant decrease in ALS and MS spinal cord MT3 levels compared to controls. This was correlated with levels of copper and MT3-copper binding, highlighting a strong relationship between copper and MT3 levels. These findings demonstrate decreased MT3 expression alongside copper as a common feature of ALS and MS supporting previously reported evidence for copper dysregulation in these diseases.

**Supplementary Information:**

The online version contains supplementary material available at 10.1038/s41598-025-31283-9.

## Introduction

Copper is vital biometal involved in the catalytic functioning of enzymes spanning a wide range of processes including radical scavenging, neurotransmitter synthesis, iron metabolism and mitochondrial respiration^1,2^. Additionally, copper also plays a role in myelin structure^3,4^ and the regulation of transcription factor activity^5^ highlighting its broad biological significance. To maintain a balance between its functional roles and potential for generation of harmful reactive oxygen species, cellular levels of copper are tightly regulated by a network of proteins responsible for its influx, efflux, transport and storage^1,2^.

While the importance of copper is broadly recognised, dysregulation of copper levels and downstream processes have also been implicated in various neurological diseases including amyotrophic lateral sclerosis (ALS) and multiple sclerosis (MS)^6,7^. ALS is predominantly characterised by the progressive death of motor neurons in the motor cortex and spinal cord grey matter, whereas MS is largely associated with central nervous system (CNS) white matter lesions connected to demyelination and axonopathy^8,9^. However, these diseases also show notable commonalities, with ALS exhibiting extensive white matter pathology^10–12^, and spinal cord grey matter deterioration being closely linked to progression of disability in MS^13,14^. In the context of copper, commonalities also exist where anatomical mapping revealed diminished copper levels in the spinal cord grey matter and an elevation localised to white matter areas associated with pathology^15,16^. Additionally, these copper changes corresponded to similar outcomes of perturbed functionality for key cuproenzymes, suggesting overlap related to altered copper processes. Although these findings indicate a shared copper dysregulation profile, whether this further extends to regulators of copper homeostasis is less clear.

Metallothioneins (MTs) are small proteins enriched in cysteine residues with a high affinity for copper and zinc, playing a key role in cellular oxidative defences by regulating metal storage and distribution^17,18^. While the MT1 and MT2 isoforms are ubiquitously expressed^19^, MT3 is primarily expressed in the CNS where it is recognised as an important isoform^18,20^. Due to its function in maintaining CNS metal homeostasis and the established link between metal dyshomeostasis and neurodegenerative diseases^6,7,21,22^, MT3 represents an important target for investigation in ALS and MS. To date, only limited examination of MT3 has been reported in ALS with histological observations^23^ and gene expression^24^ indicating decreased levels. However, quantitation of protein levels has yet to be undertaken and whether MT3 is altered in MS remains largely unexplored. In this study, we used liquid chromatography tandem mass spectrometry (LC-MS/MS) to provide robust quantitation of MT3 changes in ALS and MS spinal cord tissue against immunohistochemical assessments. This approach was supplemented with bulk inductively coupled plasma-MS (ICP-MS) and size-exclusion chromatography-ICP-MS (SEC-ICP-MS) revealing a corresponding diminution in copper levels associated with MT3.

## Materials and methods

### Human tissue

Fresh-frozen post-mortem lumbar spinal cord tissues from human ALS, MS and control cases were collected with informed consent by the Victorian Brain Bank (Australia) and MS Society Tissue Bank (UK) then stored at −80 °C. Lumbar spinal cord tissues fixed in 10% neutral-buffered formalin and embedded in paraffin were collected from the Victorian Brain Bank as 7 μm sections cut onto microscope slides. All ALS cases used in this study were clinically diagnosed as sporadic ALS, and all MS cases as either primary progressive (*n* = 6) or secondary progressive MS (*n* = 21) (Table 1). All procedures were approved by a University of Melbourne Human Ethics Committee (Project ID: 1238124) and conducted in accordance with the Australian National Health & Medical Research guidelines and regulations.

**Table 1:** Demographic information for spinal cord tissues from control, ALS and MS cases including mean age and post-mortem interval (PMI) across groups.

	Controls	ALS	MS
Cases	11	21	27
Male	7	15	14
Female	4	6	13
Mean age ± S.D.	79.4 ± 13.1	67.2 ± 9.3	52.9 ± 11.7
Mean PMI (hours)	37.3 ± 25.5	35.4 ± 16.4	27.1 ± 14.2

### Tissue preparation for mass spectrometry analyses

Whole frozen sections of lumbar spinal cord were homogenised using a motorised polypropylene pestle in tris-buffered saline (TBS; 50 mM Tris, 150 mM NaCl, pH 7.4) containing 0.5% (v/v) phosphatase inhibitor cocktail 2 (P5726, Sigma), 2% (w/v) protease inhibitors (Complete EDTA-free, 05056489001, Roche) and 5% (v/v) DNase (D5025, Sigma). Tissue homogenates were subjected to centrifugation (20,000 x g, 30 min, 4 °C) and supernatant collected as a TBS-soluble fraction and stored at −80 °C until used. Protein concentrations were determined by BCA assay (Pierce, Thermo Scientific) and then normalised using the homogenisation buffer described above.

### Liquid chromatography-tandem mass spectrometry (LC-MS/MS)

Reduction, alkylation and digestion of sample in preparation for protein identification was performed as previously described^20^. Briefly, TBS-soluble fractions were digested overnight with trypsin and endopeptidase-LysC (Tryp/LysC) in ammonium bicarbonate buffer (100 mM, pH 8.5). Synthetic peptide standards containing heavy isotopes of arginine and/or lysine were synthesized by JPT peptide technologies and Vivitide (formerly New England Biolabs), quantified by amino acid analysis, and supplied as ≥ 95% purity. These heavy peptides (750 fmol/sample) were added to digested samples and the peptides desalted using Oasis HLB µElution plates (186001828BA, Waters Corporation). Dried peptides were reconstituted to 1 mg/mL protein content in 2% v/v acetonitrile containing 0.05% v/v TFA and placed in the autosampler at 4 °C for immediate analysis. Peptides were separated using an Agilent 1290 Infinity II UPLC system fitted with an AdvanceBio Peptide Map column (150 mm x 2.1 mm, 2.7 μm particle size, 653750-902, Agilent). A gradient LC elution program was utilized for mobile phases consisting of 0.1% formic acid (0.1% v/v) in ultrapure water (buffer A), and acetonitrile plus 0.1% (v/v) formic acid (buffer B) at a flow rate of 0.4 mL/min. An Agilent 6495 triple quadrupole mass spectrometer was used to determine target peptide quantities via selected reaction monitoring (SRM). Target peptides and their associated precursor and product ions (Supplementary Fig. S1) were selected in Skyline version 20.2^25^, using the human reference proteome accessed from the UniProt database (ID: UP000005640). Retention time windows and reaction cell collision energies were optimized using the Agilent Automator add-in software for Skyline.

### Inductively coupled plasma mass spectrometry (ICP-MS)

Elemental measurements were made using an Agilent 7700 inductively coupled plasma-mass spectrometer (ICP-MS). TBS-soluble fractions were introduced via the integrated autosampler and peristaltic pump (I-AS, Agilent Technologies), connected to a glass nebulizer (MicroMist AR35-1-FM04EX, Glass Expansion, Australia). The instrument was calibrated using certified reference standards in 1% HNO_3_ (Multielement Calibration Standard 2 A, Agilent Technologies), with 100 µg/L yttrium (Y) introduced online as the internal standard (Agilent Technologies). The system hardware was operated in He gas mode (3.0 mL/min flow rate) and tuned using a commercially available stock tuning solution according to manufacturer recommendations (Agilent Technologies).

### Size-exclusion chromatography ICP-MS (SEC-ICP-MS)

Analysis of tissue metalloprotein profiles was performed as previously described^26^, with minor modifications. Briefly, TBS-soluble fractions were chromatographically separated using a Bio SEC-3 column (4.6 × 300 mm, 3 μm, 5190 − 2508, Agilent), operated at 25 °C. A mobile phase of ammonium nitrate (200 mM, pH 7.5) containing 50 ppb antimony chloride as the internal standard was delivered at a constant flow rate of 0.4 mL/min. Effluent from the in-line UV detector (Agilent 1260 HPLC), monitoring A_280_ nm, was connected to the ICP-MS nebuliser (MicroMist AR35-1-FM04EX, Glass Expansion, Australia) via PEEK tubing (0.12 mm I.D.), connected directly to the spray chamber nebuliser (MicroMist AR35-1-FM04EX, Glass Expansion, Australia). Sample injections (10 µL, containing 100 µg protein) were monitored for elemental counts over a 15.5 min period. ICP-MS signals (time-resolved elemental counts per second) were normalised to protein concentrations using a ratio of the average UV signal (A_280_) for the cohort divided by the UV signal for each sample. The ICP-MS was tuned and calibrated according to manufacturer instructions using multi-elemental calibration standards and yttrium (^89^Y) as a certified internal standard (Accustandard). The ICP-MS was operated in He mode (3.0 mL/min flow rate) and for measurement of ^56^Fe, ^63^Cu and ^66^Zn. Bovine erythrocyte SOD1 (S5395, Sigma), human liver ferritin (F6754, Sigma) and rabbit liver MT2 (Sigma) standards were also run for comparison against tissue samples. For protein identification experiments, SEC fractions were collected at 15 s intervals as 100 µL volumes into polypropylene microplates at 4 °C using an automated fraction collector (1260 Infinity II FC, Agilent Technologies). Fractions were then prepared and target proteins quantified as described in the LC-MS/MS methods.

### Immunohistochemistry

Spinal cord sections were de-paraffinised with xylene prior to rehydration through graded ethanol solutions. Slides were washed with water prior to heat-induced antigen retrieval using sodium citrate buffer containing 10 mM trisodium citrate and 0.05% (v/v) Tween 20, pH 6. After cooling at room temperature, slides were washed with water and stained using Anti-Rabbit HRP-DAB Cell and Tissue Staining Kit (CTS005, R&D Systems) following the manufacturer’s instructions. In brief, sections were blocked with sequential peroxidase, serum, avidin and biotin blocking reagents before incubation with the anti-MT3 antibody overnight at 4 °C (1:300; GTX60188; GeneTex). Sections were washed and incubated with biotinylated secondary antibody followed by streptavidin-HRP and then DAB chromogen solution. Upon DAB development, slides were washed with distilled water and cover slipped using mounting media and left to dry prior to imaging using a Mirax Digital Slide Scanner and examination using CaseViewer (3DHISTECH).

## Statistical analyses

Statistical analyses were performed using GraphPad Prism Version 10 with comparison of means undertaken. Datasets comparing groups were assessed for outliers and normality with statistical significance between groups determined using the ordinary one-way ANOVA with Šídák’s multiple comparisons test. Correlation strength was assessed using the Pearson correlation coefficient (r) and coefficient of determination (r^2^) with statistical significance reported. Lines of best fit were generated using simple linear regression analysis. Statistical significance was determined as *p* < 0.05.

## Results

**Fig. 1:**
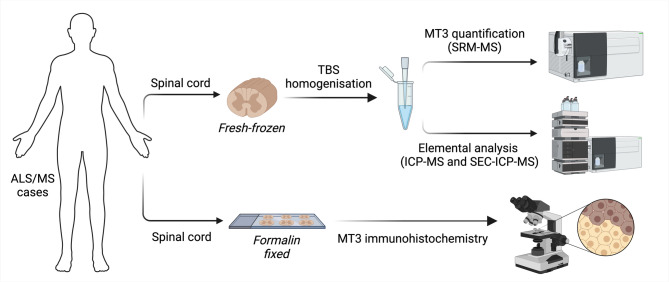
Outline of study. Fresh-frozen or formalin-fixed lumbar spinal cord from ALS, MS and control cases were homogenised or sectioned onto microscope slides, respectively. TBS-soluble fractions were then subjected to LC-MS/MS for MT3 protein quantitation, ICP-MS for elemental analysis or SEC-ICP-MS to examine protein-metal binding. Immunohistochemistry was performed on slides to qualitatively visualise localised changes to spinal cord MT3. Image generated in BioRender.

**Fig. 2:**
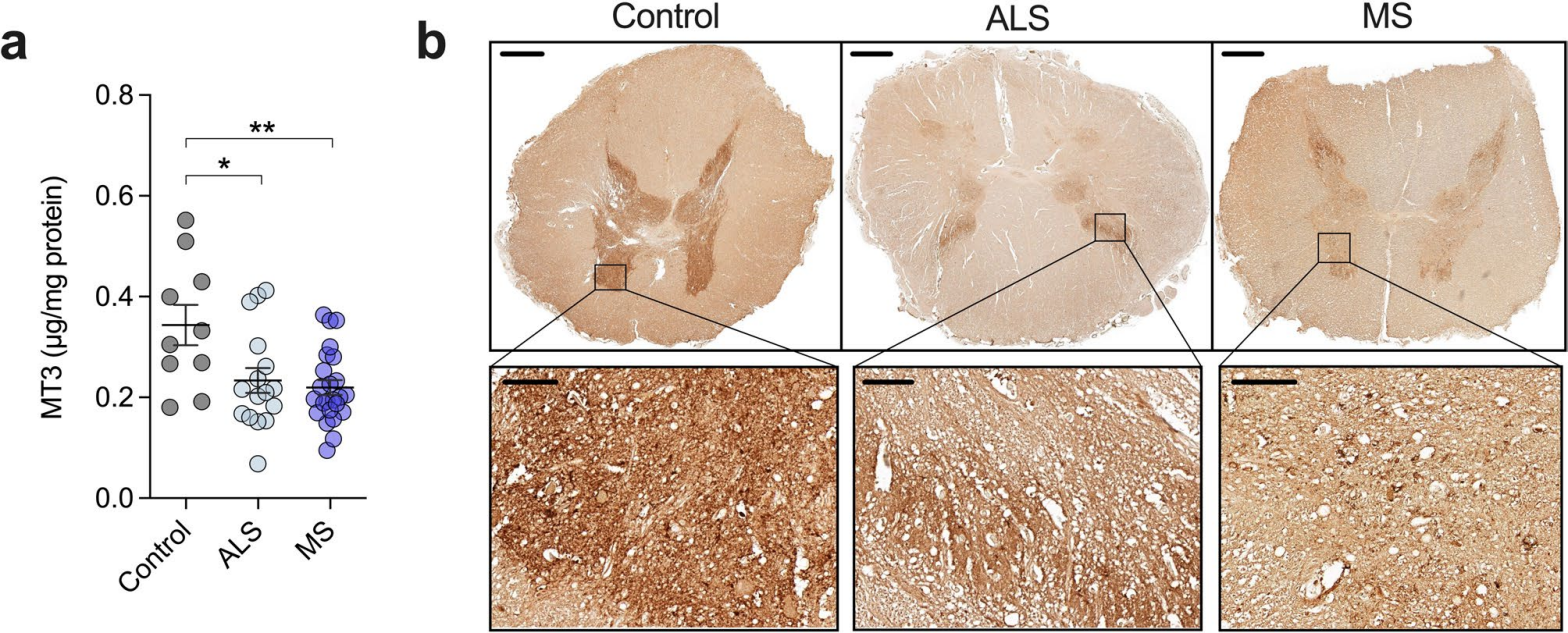
MT3 protein levels in the ALS and MS spinal cord. (**a**) Quantitative measurement of ALS and MS spinal cord MT3 levels in TBS-soluble fractions determined using LC-MS/MS and compared to control cases. (**b**) Sections of formalin-fixed spinal cord stained with MT3 using DAB as a chromogen. Higher magnification images focused on the ventral horn grey matter of spinal cord sections. Circles in (**a**) correspond to individual cases (control *n* = 10, ALS *n* = 16, MS *n* = 24) and graph presented as mean ± S.E.M. with asterisks indicating statistical significance from one-way ANOVA with Šídák’s multiple comparisons test (* *p* < 0.05; ** *p* < 0.01). Black scale bars represent 1 mm for lower magnification images and 100 μm for higher magnification images.

Amongst its various reported biological functions, the most well-characterised and prominent role of MT3 is centred around maintaining the homeostatic balance of copper and zinc^18^. To investigate alterations to MT3 in the human ALS and MS spinal cord, and how these relate to levels of copper and zinc, we applied parallel mass spectrometry approaches in conjunction with immunohistochemistry (Fig. 1). Initially, spinal cord tissue samples were measured for MT3 levels using a previously reported LC-MS/MS workflow^20^. This technique is highly sensitive and selective for targeted protein isoforms, providing an advantage over semi-quantitative immunodetection methods such as western blotting^27^. Protein levels of MT3 were found to be significantly decreased for both the ALS (32%, *p* = 0.0154) and MS (36%, *p* = 0.0027) spinal cord (Fig. 2a) in agreement with previous measurement of the MT3 transcript in MS^24^. To investigate whether other MT isoforms showed alterations, we quantified MT1 and MT2 levels by LC-MS/MS, however, these were found to be unchanged in both the ALS and MS spinal cord (Supplementary Fig. S2) suggesting changes were selective for MT3.

Immunohistochemical assessment of ALS and MS lumbar spinal cord sections also showed weaker MT3 staining compared to controls which was most evident in the grey matter (Fig. 2b). This supports previous studies showing the most intense MT3 immunohistochemical staining arising in the spinal cord grey matter where it is prominently decreased in ALS cases^23^, in addition to a more pronounced decrease in MT3 mRNA levels in the MS spinal cord grey matter^16^. Together, these results provide quantitative evidence for decreased MT3 in the ALS and MS spinal cord and further support for this decrease occurring primarily within the grey matter.

**Fig. 3:**
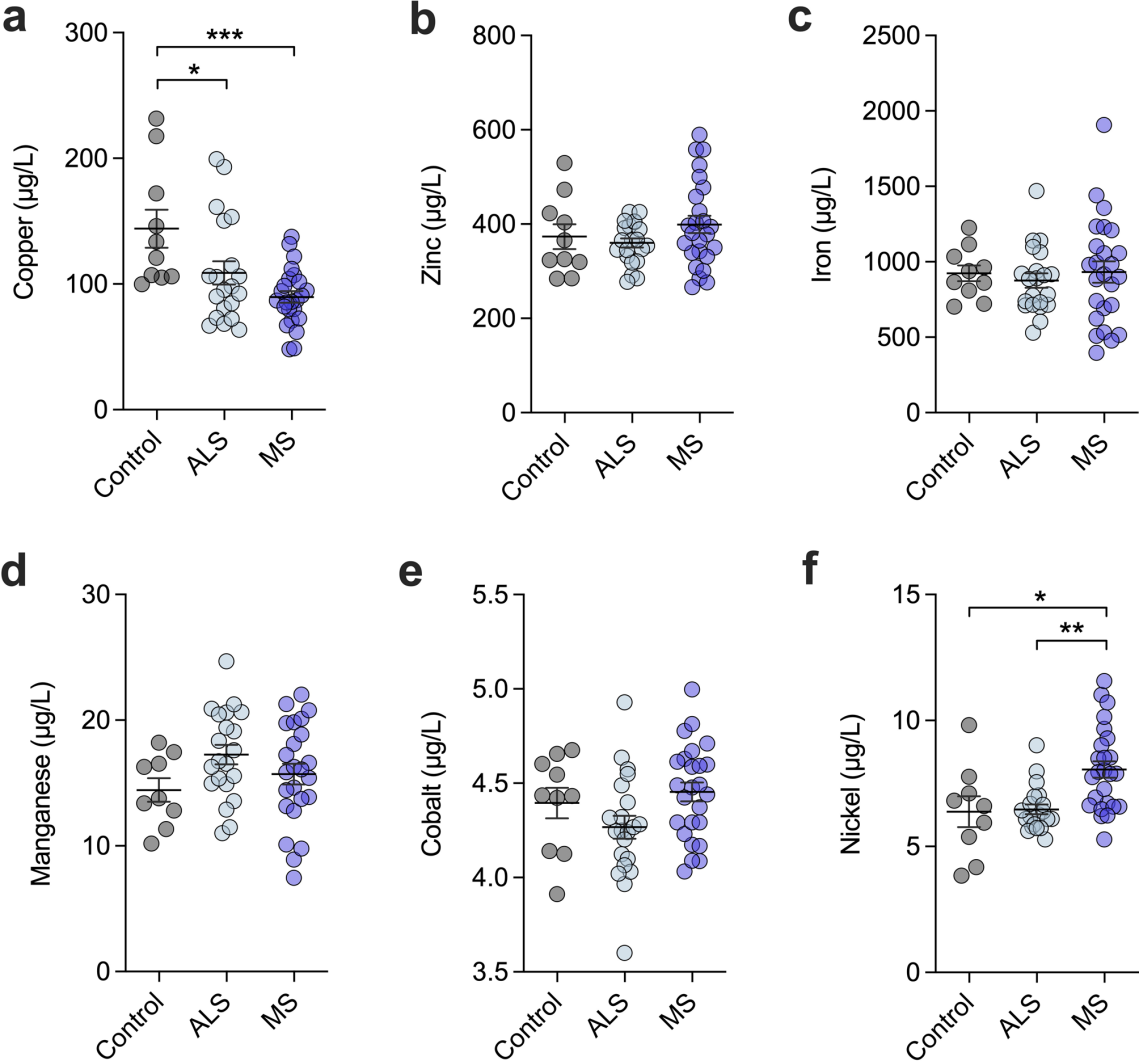
Elemental analysis of the ALS and MS spinal cord. (**a-e**) ICP-MS performed to measure levels of copper (**a**), zinc (**b**), iron (**c**), manganese (**d**), cobalt (**e**) and nickel (**f**) in TBS- soluble spinal cord fractions compared to control cases. Circles correspond to individual cases (control *n* = 9–10, ALS *n* = 20–21, MS *n* = 24–25) and graph presented as mean ± S.E.M. with asterisks indicating statistical significance from one-way ANOVA with Šídák’s multiple comparisons test (* *p* < 0.05; ** *p* < 0.01; *** *p* < 0.001).

Investigations of copper and zinc levels in ALS and MS have largely been confined to biofluids such as blood and cerebrospinal fluid, yielding conflicting results^28,29^. Here, we performed elemental analysis using ICP-MS on TBS-soluble fractions from the disease-affected spinal cord (Fig. 3a-f) which showed a significant decrease in copper levels in both ALS (25%, *p* = 0.041) and MS (38%, *p* = 0.0005) compared to control cases (Fig. 3a). Importantly, these findings corroborate what we have previously observed in the human ALS and MS spinal cord soluble fractions when applying a laser ablation ICP-MS microdroplet methodology^15,16,30^. Levels of zinc, iron, manganese and cobalt (Fig. 3b-e) did not differ significantly between groups, although nickel (Fig. 3f) was elevated in MS cases compared to controls *(p =* 0.0138) and ALS *(p =* 0.002) where increased nickel has previously been reported in the MS brain^31^.

**Fig. 4:**
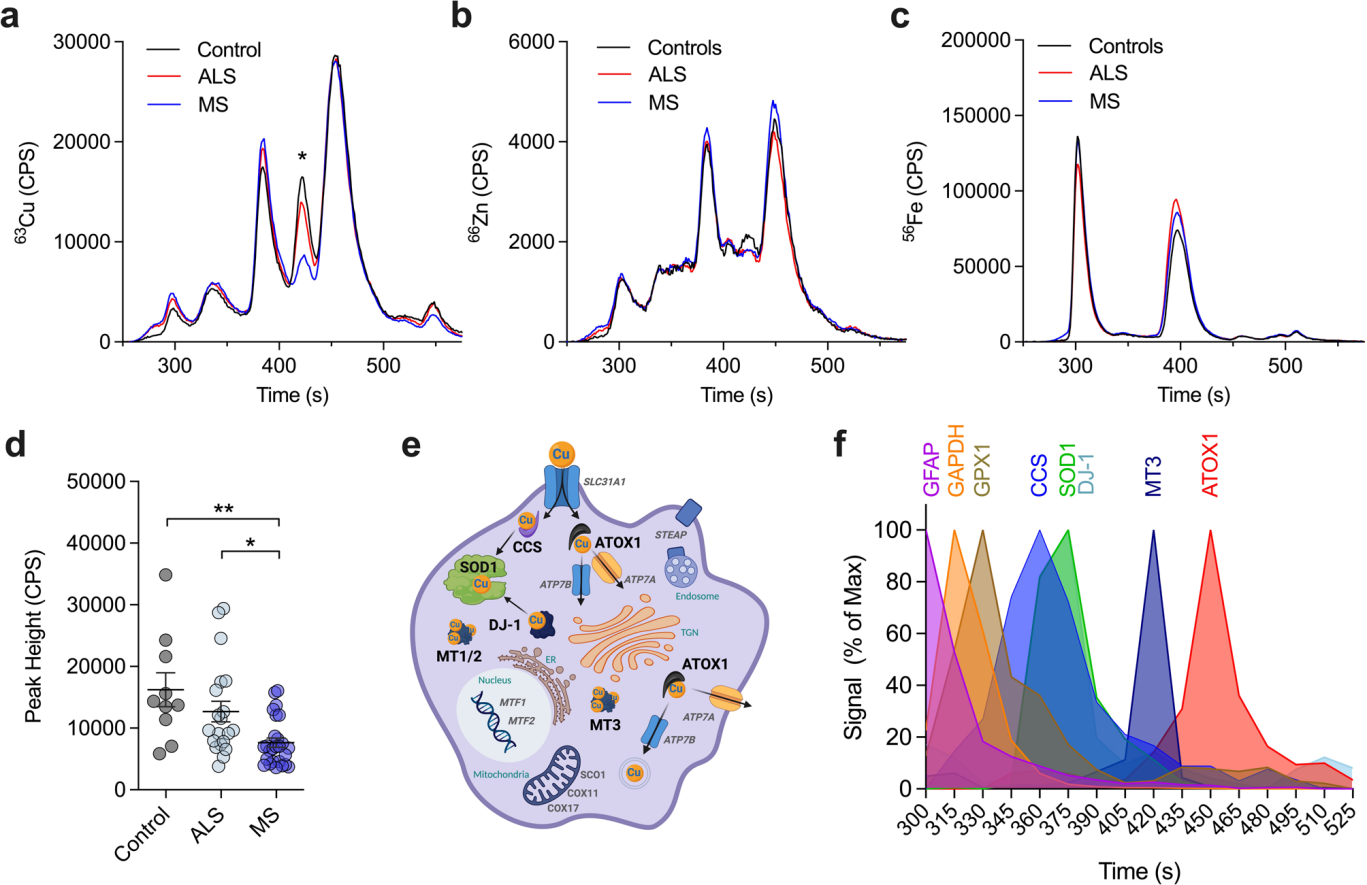
SEC-ICP-MS assessment of protein-metal binding profile. (**a-c**) Mean traces for copper as ^63^Cu isotope (**a**), zinc as ^66^Zn isotope (**b**) and iron as ^56^Fe isotope (**c**) based on elution times for ALS (*n* = 21), MS (*n* = 27) and control (*n* = 10) TBS-soluble spinal cord fractions. (**d**) Quantification of peak height for copper (**a**) at 420 s (labelled *) for ALS and MS compared to control cases. (**e, f**) Copper-binding proteins with different cellular roles (**e**) matched to SEC-ICP-MS elution times for identification of contributors to peaks in (**a-c**) using LC-MS/MS (**f**). Signal for DJ-1 overlaps with SOD1 obscuring its visualisation in (**f**). Trace lines (**a-c**) represent the mean of all cases for each group. Protein panel profiled on elution time presented as percentage relative to the maximum signal value for individual proteins collected at different elution times. Circles (**d**) correspond to individual cases (control *n* = 10, ALS *n* = 20, MS *n* = 26) and graph presented as mean ± S.E.M. with asterisk indicating statistical significance from one-way ANOVA with Šídák’s multiple comparisons test (* *p* < 0.05; ** *p* < 0.01). Image in (**e**) was generated in BioRender.

The use of chromatography coupled ICP-MS allows for characterisation of protein-metal binding profile in biological samples of interest^26^ and has been used to great effect in quantitatively matching metal traces with metalloprotein peaks^32–34^. To determine the relationship between altered MT3 and copper levels, we utilised SEC-ICP-MS on TBS-soluble spinal cord fractions from ALS and MS cases. The trace for copper demonstrated broadly consistent overlap for ALS and MS compared to controls, with the notable exception of the peak eluting at ~ 420 s showing a modest drop for ALS and a clear difference for MS (Fig. 4a). This was in contrast to the zinc and iron traces which showed similar distributions for the three groups (Fig. 4b, c). Quantification of this copper peak revealed a 22% and 53% decrease for ALS and MS, respectively, although this was statistically significant only for the MS group compared to controls *(p =* 0.0017) and ALS *(p =* 0.0276) (Fig. 4d).

To identify proteins associated with the copper peaks, TBS-soluble fractions were sampled across different SEC timepoints and measured for a panel of copper-binding proteins and reference proteins (GFAP, GAPDH, GPX1) by LC-MS/MS (Fig. 4e, f; Supplementary Fig. S1; Supplementary Fig. S3). Using this approach, MT3 was found to be the cuproprotein most associated with the 420–435 s eluate (Fig. 4f) supporting the decreased copper signal at ~ 420 s being attributable to MT3 binding. In contrast, there were two major unchanged copper peaks, with one at ~ 450 s associated with ATOX1, and the other at ~ 385 s associated with superoxide dismutase 1 (SOD1), copper chaperone to SOD1 (CCS), and the copper binding protein DJ-1^35,36^ (Fig. 4a, f; Supplementary Fig. S3). Attribution of the 385 s peak to SOD1 was also supported by the presence of a prominent zinc peak at this timepoint, reflecting the native copper/zinc binding of the SOD1 holoenzyme (Fig. 4b), as well as elution of the holo-SOD1 standard at the same time (Supplementary Fig. S4). Interestingly, MT1 and MT2 signals were below limit of quantitation measured by LC-MS/MS following SEC fraction collection (Supplementary Fig. S3g). A possible explanation for this is that the target peptide sequence used for MT1 and MT2 LC-MS/MS detection is cysteine rich (Supplementary Fig. S1) and prone to oxidation during SEC fraction collection impacting subsequent LC-MS/MS analysis. However, direct SEC-ICP-MS measurement of copper and zinc traces using a MT2 standard showed prominent peaks at ~ 450 s (Supplementary Fig. S4d, e) matching those observed at ~ 450 s for the human spinal cord. In addition, previous SEC-ICP-MS analysis has shown that MT1 and MT2 elute together and have a longer retention time compared to MT3^37^ supporting association with the notable copper and zinc peaks observed at ~ 450 s. Together, this substantiates MT3 as the source of the decreased 420 s peak for copper.

**Fig. 5:**
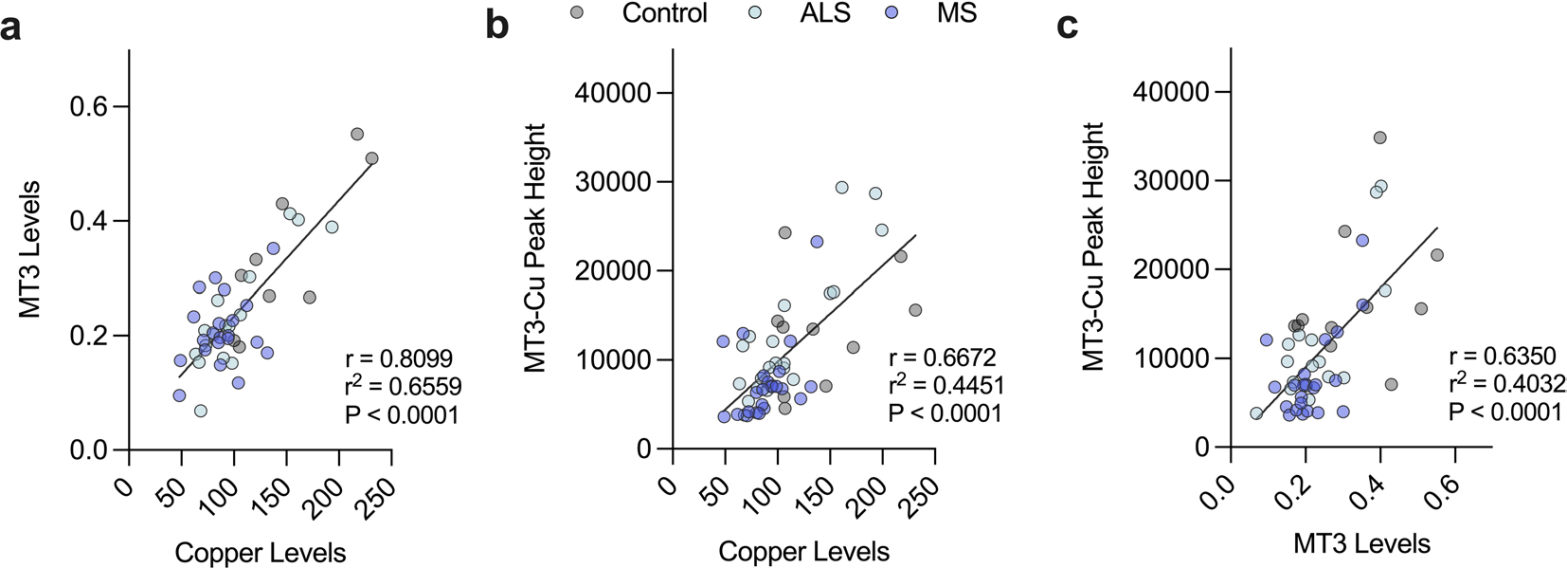
Correlation analyses of MT3, copper and MT3 associated copper peak at 420 s. (**a-c**) Plots for MT3 levels vs. copper levels (**a**), MT3-copper peak vs. copper levels (**b**) and MT3-copper peak vs. MT3 levels (**c**) show strong positive correlations. Strength of relationship assessed using the Pearson correlation coefficient (r) and coefficient of determination (r)^2^. Cases without values for both variables were excluded from analyses. Circles correspond to individual cases (control *n* = 9, ALS *n* = 16–20, MS *n* = 22–24) with graphs showing the line of best fit using simple linear regression and statistical significance determined as *p* < 0.05.

Having observed decreases relating to MT3 levels (Fig. 2a) and copper (Fig. 3a) in the human ALS and MS spinal cord, correlation analyses were performed to determine the strength of the relationship between them (Fig. 5). The strongest correlation was seen between MT3 and copper levels (Fig. 5a), with the 420 s copper peak associated with MT3 (Fig. 4d) also being strongly correlated with copper levels (Fig. 5b) and MT3 levels (Fig. 5c). In contrast, negligible or weak correlations were observed between these variables and the age of cases used, although these were found to be stronger for MT3 levels (Supplementary Fig. S5). Taken together, these results provide support for a connection between MT3 and copper in the human ALS and MS spinal cord highlighting another point of commonality between the two diseases.

## Discussion

The importance of copper as a biological metal is underscored by its many roles, however, its limited natural turnover rate in the CNS^38^ illustrates the vulnerability of this region to copper dysregulation in pathology. Copper overload is a defining feature of Wilson’s disease where mutations to the copper transporter ATP7B lead to impaired copper efflux and resultant accumulation in multiple organ systems including the CNS^39^. This excess of copper is then thought to drive oxidative stress and damage^39^ with mechanisms triggering apoptosis and the newly described cuproptosis also being implicated^40,41^. Elevated copper in Alzheimer’s disease has also been widely reported^42^, although evidence exists to show that while elevated copper is associated with plaques^43^, brain copper levels more broadly are decreased^44,45^ and improving copper bioavailability can ameliorate pathology in a mouse model of disease^46,47^.

On the other hand, insufficient copper levels are involved in various disorders of the CNS such as Menkes disease which is characterised by systemic copper deficiency and cuproenzyme dysfunction due to ATP7A mutations^48,49^. Outside of genetic causes, copper has been found to be decreased in the Parkinson’s disease brain corresponding to diminished cuproenzyme metallation and function linked to pathology^50–54^. In rare cases deficiency can be acquired through copper malabsorption resulting in copper deficiency myelopathy that presents with myelin abnormalities, axonal damage and white matter lesions^55–57^.

In the context of ALS, a key role for copper has been considered since the discovery of mutations to *SOD1* as the first genetic cause of ALS and responsible for encoding the copper and zinc binding metalloenzyme SOD1^58^. While the impact of SOD1 mutations on metal status and function was initially confined to a subset of mutants^59^, this has been expanded to mutants considered ‘wild-type like’^60^. These SOD1 mutants still possess similar metal binding capacity and enzymatic activity under cell-free conditions^59^ but display evidence of copper deficiency linked to protein misfolding and aggregation events observed in ALS^61–66^. Copper alterations also extend beyond SOD1 affecting both copper homeostasis^67–69^ and other cuproenzymes^70^ which can be recovered through overexpression of the transporter CTR1 required for cellular copper influx^63,70^. Additionally, evidence for alterations to copper and cuproenzyme function in human sporadic ALS^15^ suggests that copper dysregulation in ALS is not just limited to familial cases connected to SOD1 mutations.

Despite the known link between copper deficiency and myelin abnormalities^55–57^, as well as the use of the copper chelator cuprizone as a model of MS demyelination^71^, copper has remained underexplored in MS. Although the mechanism by which cuprizone exerts its effects remains controversial^72,73^, it has been recently shown that cuprizone decreases copper in affected CNS areas and is recovered with improved copper bioavailability^74^. The relevance of copper in MS has also been shown in the experimental autoimmune encephalomyelitis (EAE) model and human MS cases which exhibit an upregulation of the copper transporters ATP7A, ATP7B and CTR1 in white matter regions associated with lesions^75^. Moreover, copper perturbations have been found to be pervasive across the MS spinal cord and correspond to alterations in copper related gene expression and cuproenzyme functioning^16^ demonstrating a broader copper impact in MS.

Being a key element of copper metabolism, metallothioneins, and in particular the CNS-selective MT3 isoform^18,20^, represent a logical point of investigation for neurological diseases associated with copper. Interestingly, MT3 levels in the mutant SOD1 mouse model of ALS were reportedly elevated^76–78^, however, knocking out MT3 worsened phenotype and survival^79^, and retrograde viral delivery of MT3 was found to be beneficial^80^. Taken together, these findings provide support for MT3 having a protective role in ALS. In contrast, only limited evidence exists for MT3 in MS with mRNA levels being unchanged in EAE mice and decreased in the human grey matter^16^. Here, we found that MT3 protein levels were decreased in both the ALS and MS spinal cord, with the former observation supporting reports of diminished gene expression and immunohistochemical staining^23,24^. Why spinal cord levels of MT3 appear to be elevated in SOD1 mice but decreased in human ALS remains to be determined. However, it is conceivable that this is due to mutant SOD1 overexpression driving an elevated demand for copper^64,68,78^, which although inadequately met^64^, could affect expression levels of MT3 in this model.

Copper levels were also observed to be selectively decreased in the ALS and MS spinal cord showing a strong correlation with MT3 levels. In terms of copper binding to MT3, SEC-ICP-MS analysis showed a significant drop in copper signal associated with the peak corresponding to MT3 for the MS spinal cord, and this strongly correlated with copper and MT3 levels for both ALS and MS. For this study, we used TBS-soluble spinal cord fractions required for performing SEC-ICP-MS analysis^26,81^ and to maintain consistency across the methodologies used. The TBS-soluble fraction accounts for the majority of total CNS copper^15,16,44,82^ and is highly enriched for proteins in the cytoplasm^16,74^, where MT3 is primarily located. However, this approach excludes TBS-insoluble material which has been found to show opposing changes in copper levels in the ALS and MS spinal cord^15,16^. As a result, the analyses reported here are restricted to the TBS-soluble fraction where other changes could be present in the TBS-insoluble fraction. Additionally, although prominent cuproproteins were included in our SEC-ICP-MS elution profiling, we cannot exclude the possibility of other unidentified cuproproteins contributing to the decreased copper peak observed.

Similar to MT3, elevated levels of MT1 and MT2 have been observed in the mutant SOD1 mouse model^76,78,83^, with MT1 and MT2 depletion exacerbating disease phenotype^79,84^ while their induction was protective^85^. MT1 and MT2 expression in human ALS, however, is less clear, with both increased^86^ and decreased^23^ immunoreactivity having been reported. In the context of MS, limited investigations have been undertaken though increased MT1 and MT2 immunoreactivity has been observed in cuprizone-treated mice^87^. Additionally, MT2 treatment caused a modest attenuation of EAE disease phenotype^88^, while MT1/MT2 gene expression was found to be elevated in spinal cords of EAE mice and human MS cases^16^. While these findings indicate MT1 and MT2 involvement in ALS and MS, here, we found no difference in MT1 and MT2 levels for the human ALS and MS spinal cord suggesting divergent impacts on specific MT isoforms.

Although copper and MT3 levels were found to be strongly correlated and significantly decreased in both the ALS and MS spinal cord, the implications of this remain to be understood. Notably, it is reported that MT3 expression is not directly responsive to metal ions such as copper which might be explained by the uniqueness of its sequence and structure^18,89,90^. This contrasts with MT1 and MT2 which possess several metal-responsive elements in the gene promoter regions allowing for binding of metal regulatory transcription factor-1 (MTF1) in response to activation by metal ions^91,92^. This distinction in transcriptional regulation, and the absence of a change to MT1 and MT2, suggests that decreased MT3 levels may not necessarily be a direct regulatory result of decreased copper levels but reflect parallel disease processes or broader copper dysregulation previously reported in the human ALS and MS spinal cord^15,16^. It is plausible that glutathione changes in the ALS and MS spinal cord could influence copper binding to MT3, given the complex interplay between glutathione and metallothioneins in cellular copper homeostasis based on redox activity^93,94^. This possibility is also supported by reports of diminished glutathione levels in both human ALS^95,96^ and MS^97^. However, whether glutathione impacts MT3 copper binding in ALS and MS, and why MT1 and MT2 would appear to remain unaffected, remain open questions for further investigation. While unfeasible in this study due to the exhaustion of available samples, future research would be valuable to examine glutathione levels in conjunction with copper and MT levels, TBS-insoluble fraction changes and differences between white and grey matter to more comprehensively disentangle their role in ALS and MS.

Improving our understanding of copper-related processes in neurological diseases such as ALS and MS is important for determining the extent to which therapeutic strategies targeting them might work. The utilisation of a copper chelation approach in mutant SOD1 mice has previously shown benefit^68,98^, and may exert its effects through attenuating levels of partially metallated and less stable zinc-deficient SOD1^60^ which has been implicated in nitric oxide-dependent cell toxicity^99,100^. Conversely, therapeutic improvement of SOD1 copper metallation has also shown the ability to significantly improve phenotype and survival of mutant SOD1 mice^63,101,102^ and could be applicable to the human sporadic form of ALS which shows evidence of copper dysregulation unrelated to SOD1 mutations^15^. Similarly, in MS it has been proposed that aberrant copper redistribution from astrocytes to neighbouring CNS cells due to neuroinflammation in white matter lesions could be targeted therapeutically^75^. Although anatomical imaging revealing elevated white matter copper levels lends support to this approach, diminished grey matter copper and cuproenzyme function in MS^16^ highlights the need for a holistic view of copper dysregulation.

Despite ALS and MS being clinically distinct neurological diseases with differing aetiologies and pathological manifestations, conspicuous overlap exists. In particular, copper dysregulation is emerging as a potential common thread amenable to therapeutic intervention. Here, we identified decreased MT3 and copper as highly correlated features of the human ALS and MS spinal cord, providing further support for copper-related mechanisms being a unifying feature of both diseases.

## Supplementary Information

Below is the link to the electronic supplementary material.

Supplementary Material 1

## Data Availability

The underlying data in this work are presented in the article and can be made available upon request.

## Abbreviations

ALS: amyotrophic lateral sclerosis
ANOVA: analysis of variance
ATOX1: antioxidant 1 copper chaperone
ATP7A: ATPase copper transporting alpha
ATP7B: ATPase copper transporting beta
BCA: bicinchoninic acid
CCS: copper chaperone for superoxide dismutase
CNS: central nervous system
CPS: counts per second
CTR1: copper transporter 1
Cu: copper
DAB: 3,3’-diaminobenzidine
DJ-1: Parkinson disease protein 7 (PARK7)
EAE: experimental autoimmune encephalomyelitis
EDTA: ethylenediaminetetraacetic acid
Fe: iron
GAPDH: glyceraldehyde-3-phosphate dehydrogenase
GFAP: glial fibrillary acidic protein
GPX1: glutathione peroxidase 1
He: helium
HLB: hydrophobic-lipophilic balanced
HNO_3_: nitric acid
ICP-MS: inductively coupled plasma mass spectrometry
LC: liquid chromatography
HPLC: high-performance liquid chromatography
HRP: horseradish peroxidase
mRNA: messenger ribonucleic acid
MS: multiple sclerosis
MS/MS: tandem mass spectrometry
MT1: metallothionein-1
MT2: metallothionein-2
MT3: metallothionein-3
MTF1: metal regulatory transcription factor-1
SEC: size exclusion chromatography
S.E.M.: standard error of the mean
SOD1: superoxide dismutase 1
SRM: selected reaction monitoring
TBS: tris-buffered saline
TFA: trifluoroacetic acid
UPLC: ultra-performance liquid chromatography
UV: ultraviolet
Y: yttrium
Zn: zinc
