# Supplementary material for: Decreased metallothionein-3 expression in the human spinal cord is a common feature of amyotrophic lateral sclerosis and multiple sclerosis
Source: Sci Rep. 2026 Mar 4;16:9598. doi: 10.1038/s41598-025-31283-9 (PMC13009280; doi:10.1038/s41598-025-31283-9)
Supplement: Supplementary file 1 — Supplementary Material 1 [file 41598_2025_31283_MOESM1_ESM.docx]

**Supplementary data:**

**Supplementary Figure 1.**

| **Protein Target(s)** | **Sequence (heavy isotope in bold)** | **Precursor m/z** | **Product m/z (Collision Energy)** | | | | |
| --- | --- | --- | --- | --- | --- | --- | --- |
| MT1 & MT2 | CAQGCICK | 498.7066++ | 836.4+ (20) | 765.3+ (17) | 637.3+ (17) | 580.3+ (17) |  |
| MT1 & MT2 | CAQGCIC**K** | 502.7137++ | 844.4+ (20) | 773.4+ (17) | 645.3+ (17) | 588.3+ (17) |  |
| MT3 | GGEAAEAEAEK | 531.2409++ | 818.4+ (18) | 747.4+ (18) | 676.3+ (18) | 347.2+ (27) |  |
| MT3 | GGEAAEAEAE**K** | 535.248++ | 826.4+ (18) | 755.4+ (18) | 684.3+ (18) | 355.2+ (27) |  |
| Park7 (DJ-1) | DVVICPDASLEDAK | 766.3691++ | 1105.5+ (25) | 945.5+ (25) | 215.1+ (25) | 314.2+ (25) | 427.3+ (25) |
| GPX1 | CEVNGAGAHPLFAFLR | 586.9629++ | 863.5+ (16) | 653.4+ (16) | 685.9++ (16) | 628.8++ (16) | 432.3++ (16) |
| GFAP | DNLAQDLATVR | 608.3200++ | 873.5+ (20) | 802.4+ (20) | 674.4+ (20) | 230.1+ (20) | 343.2+ (20) |
| GAPDH | GALQNIIPASTGAAK | 706.3988++ | 1042.6+ (23) | 815.5+ (23) | 702.4+ (23) | 242.1+ (23) | 597.3+ (23) |
| CCS | SLIIDEGEDDLGR | 716.3517++ | 1118.5+ (23) | 1005.4+ (23) | 890.44+ (23) | 761.3+ (23) | 345.2+ (23) |
| SOD1 | TLVVHEK | 275.8325+++ | 512.3+ (5) | 413.2+ (5) | 362.7++ (5) | 306.2++ (5) |  |
| ATOX1 | YDIDLPNK | 489.2506++ | 699.4+ (16) | 586.3+ (16) | 471.3+ (16) | 358.2+ (16) | 279.1+ (16) |
|  |  |  |  |  |  |  |  |
|  | | |  |  |  |  |  |

***Supplementary figure 1.*** *Overview of peptides used for LC-MS/MS identification of target proteins with their associated precursor/product ion masses (Da) and charge states (indicated by number of ‘+’ symbols). Energies (eV) used to generate product ions by collision-induced dissociation (CID) are detailed in parentheses.* *Cysteine residues (C) were monitored as the carbamidomethyl form (+57.021464 Da). Heavy isotope labelled lysine residues (^13^C-lysine, +8 Da) are indicated with ‘K’ in boldface.*

**Supplementary Figure 2.**

**
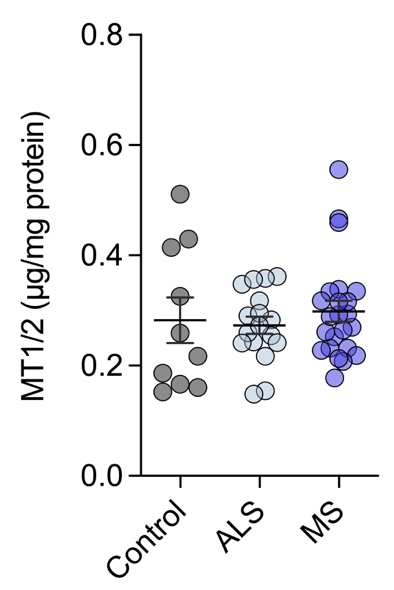
**

***Supplementary figure 2.*** *MT1/2 protein levels in the ALS and MS spinal cord TBS-soluble fraction quantified using LC-MS/MS and compared to control cases. Circles correspond to individual cases (control n=10, ALS n =17, MS n=23) and graph presented as mean ± S.E.M.*

**Supplementary Figure 3.**

**
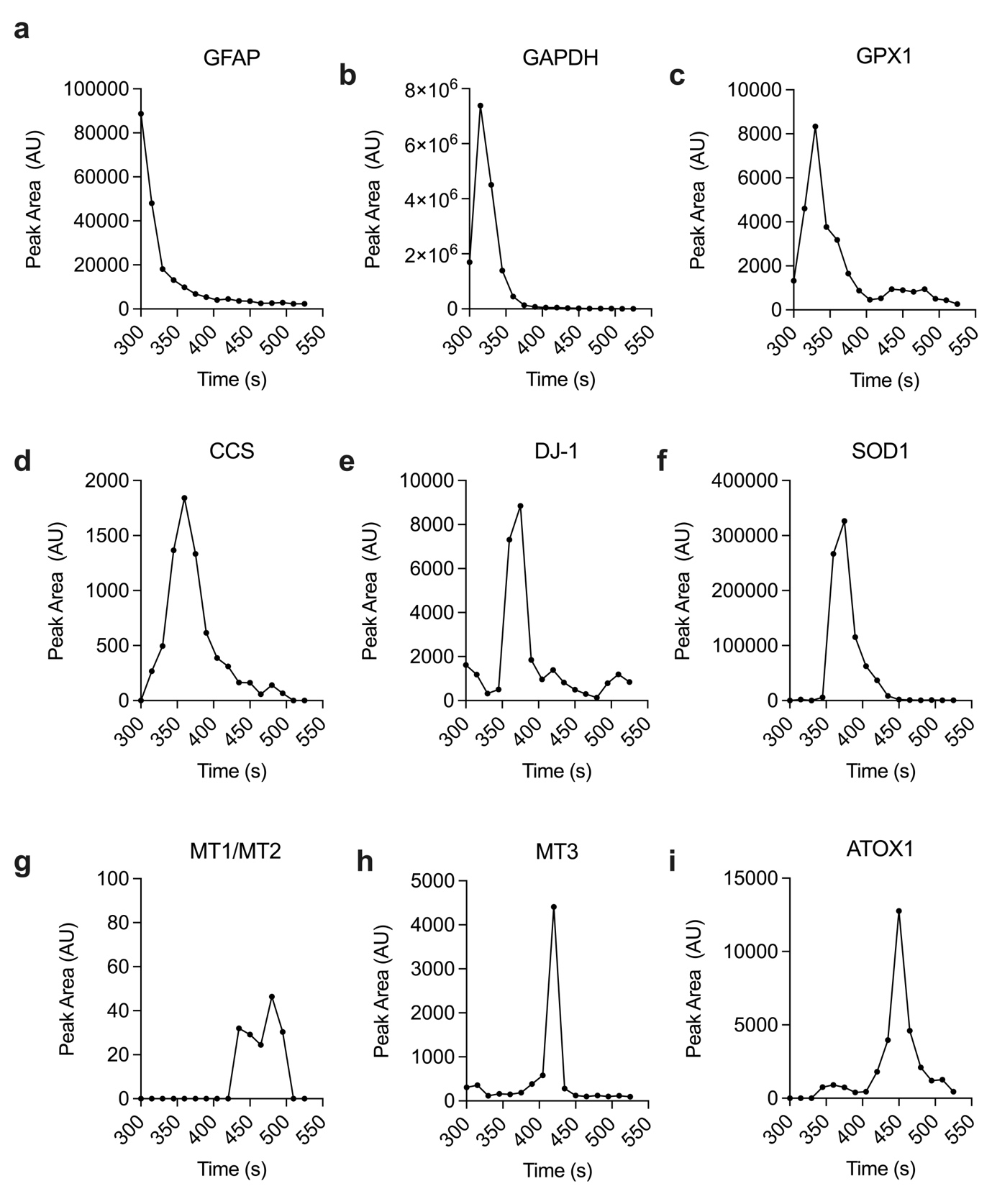
**

***Supplementary figure 3.*** *Elution profiles of selected housekeeping proteins (a-c) and endogenous CNS tissue cuproproteins (d-i) observed during size-exclusion chromatography (SEC). Analytical SEC was performed using conditions identical to the online SEC-ICP-MS workflow (see methods), with the modification of directing the column outflow to a fraction collector to sample fractions at 15 second intervals. The resulting fractions were processed for LC-MS/MS analysis by digesting overnight with trypsin, then desalting and concentrating by solid-phase extraction. Proteins were identified using selected reaction monitoring LC-MS/MS. Signals shown here (sum of peak areas) were plotted for each target against their representative elution times from the SEC column.*

**Supplementary Figure 4.**

**
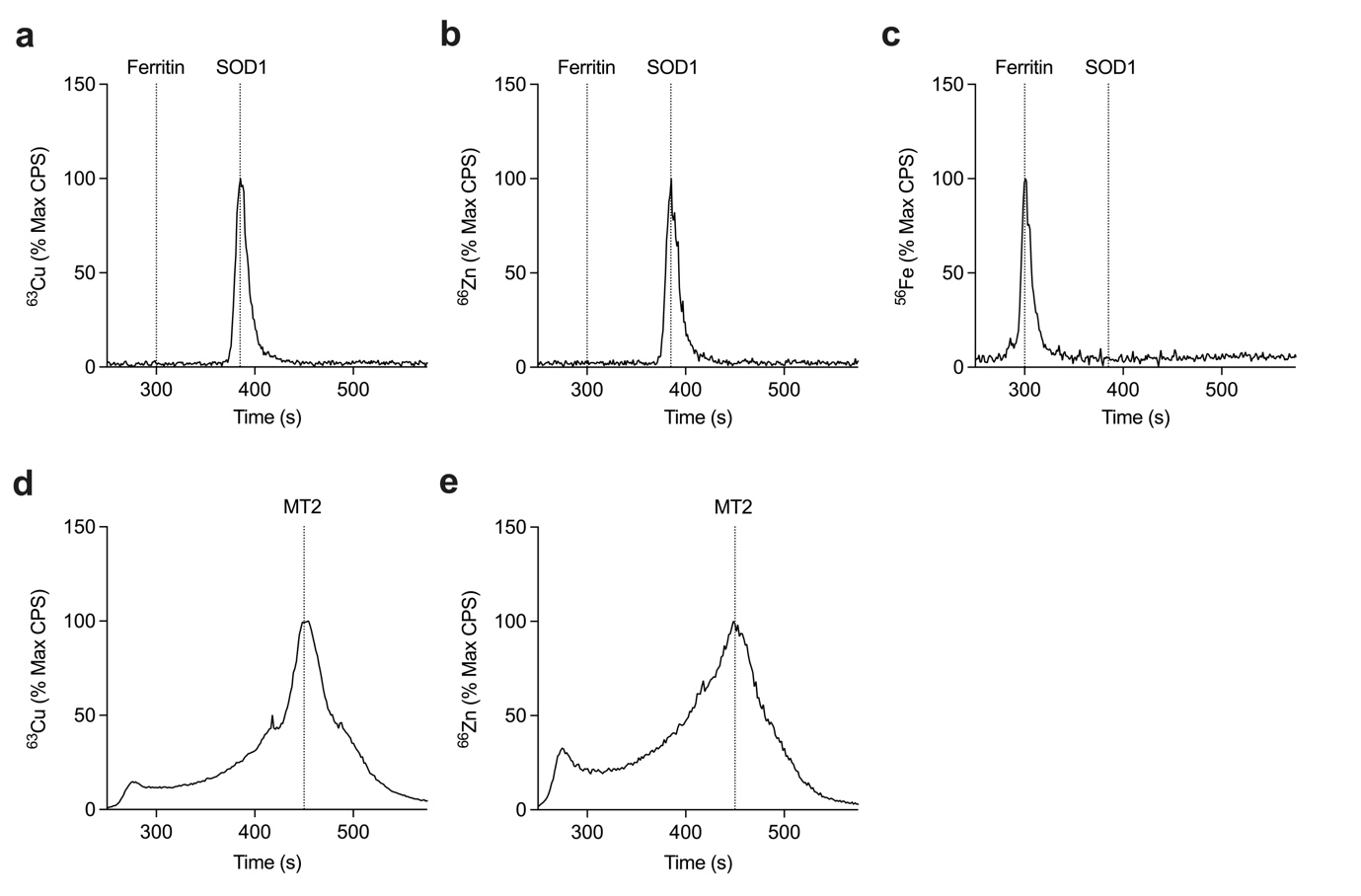
**

***Supplementary figure 4.*** *SEC-ICP-MS traces for SOD1, ferritin and MT2 standards. (a,b) Cu/Zn binding SOD1 (holo-SOD1) showed peaks at ~385 seconds for copper (a) and zinc (b) while the iron storage protein ferritin showed a peak at ~300 seconds for iron (c). Dashed lines indicate 300 seconds and 385 seconds for each trace. (d,e) MT2 showed a broader signal profile with the main peaks at ~450 seconds for copper (d) and zinc (e). Dashed lines indicate 450 seconds for each trace. Traces presented as percentages of the maximum signal measured in counts per second (CPS).*

**Supplementary Figure 5.**

**
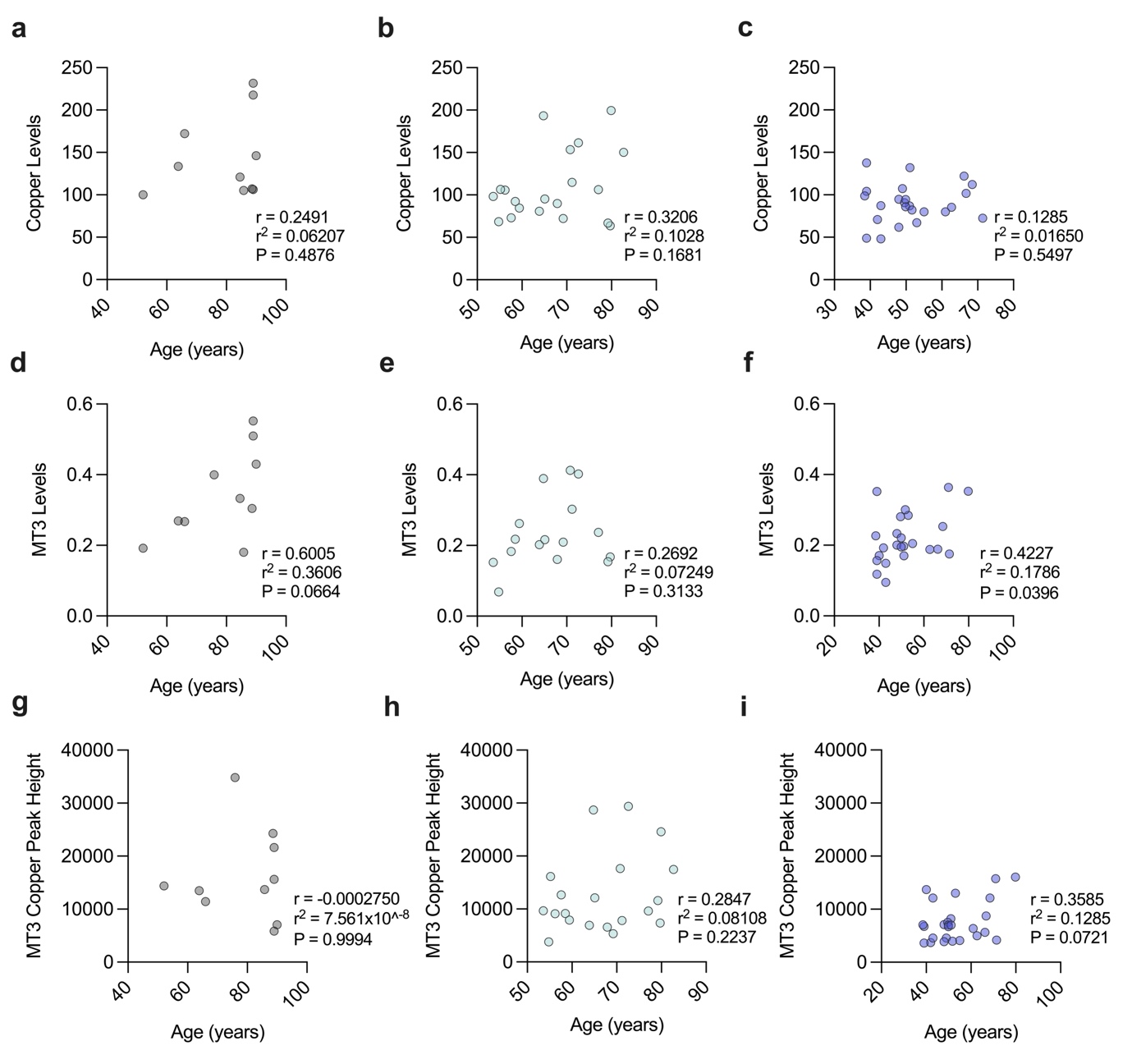
**

***Supplementary figure 5.*** *Correlation analyses for MT3, copper and MT3-copper peak against the age of ALS, MS and control cases used. (a-c) Correlation between copper levels and age for controls (a), ALS (b) and MS (c) cases. (d-f) Correlation between MT3 levels and age for controls (d), ALS (e) and MS (f) cases. (g-i) Correlation between MT3-copper peak height and age for controls (g), ALS (h) and MS (i) cases. Strength of relationship assessed using the Pearson correlation coefficient (r) and coefficient of determination (r^2^). Cases without values for both variables were excluded from analyses. Circles correspond to individual cases (control n=10, ALS n=16-20, MS n=24-26) with statistical significance determined as p<0.05.*
